# Loss of signal transducer and activator of transcription 3 impaired the osteogenesis of mesenchymal progenitor cells in vivo and in vitro

**DOI:** 10.1186/s13578-021-00685-3

**Published:** 2021-09-08

**Authors:** Zijing Huang, Jingyi Feng, Xin Feng, Laiting Chan, Jiarui Lu, Lizhen Lei, Zhuwei Huang, Xiaolei Zhang

**Affiliations:** 1 Department of Operative Dentistry and Endodontics, Hospital of Stomatology, Guanghua School of Stomatology, Sun Yat-Sen University, Guangzhou, Guangdong China; 2 Guangdong Province Key Laboratory of Stomatology, Guangzhou, Guangdong China

**Keywords:** Stat3, Bone development, Osteogenesis, BMSCs

## Abstract

**Background:**

Signal transducer and activator of transcription 3 (Stat3) is a cytoplasmic transcription factor that participates in various biologic processes. Loss of Stat3 causes hyperimmunoglobulin E syndrome, presenting with skeletal disorders including osteoporosis, recurrent fractures, scoliosis, and craniosynostosis. The objective of this study is to explore the effect and mechanism of Stat3 on osteogenesis of mesenchymal progenitors.

**Methods:**

Stat3 was conditionally knockout (CKO) in mesenchymal progenitors by crossing the pair-related homeobox gene 1-cre (Prx1-Cre) with Stat3-floxed strain mice. Whole-mount-skeletal staining, histology, and micro-CT were used to assess the differences between Stat3 CKO and control mice. Further, in vitro experiments were conducted to evaluate the osteogenesis potential of primary isolated bone marrow mesenchymal stem cells (BMSCs) from both control and Stat3 CKO mice. After osteogenic induction for 14d, alizarin red staining was used to show the calcium deposit, while the western blotting was applied to detect the expression of osteogenic markers.

**Results:**

Compared with the control, Stat3 CKO mice were present with shortened limbs, multiple fractures of long bone, and open calvarial fontanels. The abnormal growth plate structure and reduced collagen fiber were found in Stat3 CKO limbs. According to micro-CT analysis, the reduced cortical bone thickness and bone volume were found on Stat3 CKO mice. The in vitro osteogenic differentiation of BMSCs was inhibited in Stat3 CKO samples. After osteogenic induction for 14d, the significantly diminished calcium deposits were found in Stat3 CKO BMSCs. The decreased expression of osteogenic markers (OPN and COL1A1) was observed in Stat3 CKO BMSCs, compared with the control.

**Conclusions:**

Stat3 played a critical role in bone development and osteogenesis. Loss of Stat3 impaired the osteogenesis of mesenchymal progenitors in vivo and in vitro.

**Supplementary Information:**

The online version contains supplementary material available at 10.1186/s13578-021-00685-3.

## Background

The skeleton development is a complex process involving cell migration, proliferation, and differentiation [1]. The differentiation of mesenchymal progenitors into osteoblasts is critical for both intramembranous and endochondral ossification [2]. Such process requires the participation of multiple signaling pathways. The mutation or abnormal expression of relevant genes can cause serious diseases [2].

Signal transducer and activator of transcription 3 (Stat3) is a cytoplasmic transcription factor and extensively participates in cell proliferation and differentiation [3]. After tyrosine phosphorylation by cytokines and growth factors, Stat3 molecules dimerize and translocate into the nucleus and then regulate gene transcription [4].

Heterozygous Stat3 mutation in humans leads to Job syndrome (also named hyper-IgE syndrome), which is characterized by recurrent multiple organ infection along with skeletal, dental abnormalities [5–7]. The skeletal features of Job syndrome patients comprise recurrent fractures, scoliosis, and cranial-maxillofacial abnormalities [7]. Mice with homozygous mutation of the Stat3 gene died during embryonic period, suggesting the essential role of Stat3 for development [8]. Mesoderm-specific loss of Stat3 caused a campomelic dysplasia phenotype, including shortened limbs at birth, limb curvature in postnatal development and fracture of long bones [9]. Via directly regulating SRY-box 9 (Sox9) expression, Stat3 played an important role in chondrogenesis [9]. Conditional knockout of Stat3 in osteoblast showed an osteoporotic phenotype, while gp130 knockin mice (enhanced activation of Stat3) demonstrated an osteosclerotic phenotype [10]. Moreover, Stat3 disruption in osteoblasts and osteocytes significantly decreased bone strength and reduced load-driven bone formation [11]. These results suggested the important role of Stat3 in osteogenesis of osteoblastic cell lineages. Considering the fact that osteoblastic cells were differentiated from mesenchymal progenitors, the effect of Stat3 on osteogenesis of mesenchymal progenitors deserved an interesting research question to explore.

In the current study, Stat3 was conditional knockout (CKO) in mesenchymal progenitors in vivo by using the pair-related homeobox gene 1-Cre (Prx1-Cre) crossed with Stat3-floxed strain mice [12–14]. The limb and craniofacial bone of Stat3 CKO mice were compared with their littermate control. Bone mesenchymal stem cells were also isolated from the respective mouse lines to assess their osteogenesis potential. The hypothesis of this study is that loss of Stat3 impairs the osteogenesis of mesenchymal progenitors in vivo and in vitro.

## Materials and methods

### Mouse breeding

The animal work was carried out according to federal guidelines, with the approval by the Animal Ethical and Welfare Committee of Sun Yat-sen University (approval number SYSU-IACUC-2021-000101). Mice were purchased from the Jackson Laboratory (Prx1-Cre, stock no.005584 [12]; Stat3^f/f^, stock no.016923 [14]). To generate mice with Stat3 conditional knockout in limb bud and craniofacial mesenchyme, Stat3^f/f^ were crossed with the Prx1-Cre mice to generate Prx1-Cre; Stat3^f/+^ mice. Next, Prx1-Cre; Stat3^f/+^ mice were crossed with Stat3^f/f^ mice to generate Prx1-Cre; Stat3^f/f^ conditional knockout (CKO) mutant mice. The Cre-negative littermates were taken as the control of Stat3 CKO mutants. Mouse tail genotyping was performed by polymerase chain reaction (PCR) and the primer sequences for PCR were listed in Table 1.

**Table 1:** Primers for genotyping

Gene	Forward	Reverse
Prx1-cre	GCGGTCTGGCAGTAAAAACTATC	GTGAAACAGCATTGCTGTCACTT
Stat3	TTGACCTGTGCTCCTACAAAAA	CCCTAGATTAGGCCAGCACA

### Whole-mount-skeletal staining

Skeletons from neonatal (P0) mice were processed for Alizarin Red S (sigma A5533) and Alcian Blue (sigma A5268) staining to demonstrate bone and cartilage. The protocol was same as previously described [15]. Pictures were taken by Leica Microsystems (MZ10F). Leica LAS EZ software was used to measure the length of limbs and the width of sagittal sutures.

### Histology and immunohistofluorescence (IHF) staining

For histology, limbs were fixed in 10% formalin (Servicebio, Wuhan, China) for 24 h. For 8-week-old samples, 4-weeks’ decalcification by 10% tetrasodium EDTA were conducted. After dehydrated by graded ethanol and cleared in xylene, limbs were embedded in paraffin and sectioned at 4 μm thickness along the long axis. Sections were stained with safranin O/fast green, masson, hematoxylin and eosin (H&E) (Servicebio, Wuhan, China). Sections were mounted in neutral resins and then scanned by AperioAT2 scanner (Leica Microsystems). The quantification of safranin O/fast green and the Masson staining was carried out by ImageJ. Specifically, in safranin O/fast green stained sections, the area of proliferative zone and hypertrophic zone was measured respectively, which was then divided by the total area of the growth plate to obtain the relative surface area of the region of interest. In the Masson-stained sections, the blue-stained collagen fiber area was divided by total tissue area to calculate the collagen fraction. For histomorphometric, three samples per group were used. For each sample, three sections were selected. The mean value of the three slides was considered as the result of one sample.

For IHF staining, the antigen retrieval was performed in a citrate buffer (Beyotime, Shanghai, China). Sections were blocked in normal goat serum (NGS) for 1 h before incubation with primary antibodies overnight at 4 °C. The next day, sections were incubated with Alexafluor-594 conjugated secondary antibody (Beyotime, Shanghai, China) for 1 h at room temperature. Sections were mounted in mounting medium with DAPI from Vector Laboratories. The images were captured by an inversion fluorescence microscope (Zeiss, Oberkochen, Germany). The antibodies used for IHF were listed in Table 2.

**Table 2:** Antibodies for flow cytometry, western blot and immunohistofluorescence staining

Antibody raised against	Dilution	Source (cat. NO)
Flow cytometry antibody		
PE Rat IgG2b, κIsotype Control	1:200 (F)	BD (553989)
FITC Rat IgG2b, κIsotype Control	1:200 (F)	BD (554688)
PE Hamster Anti-Mouse CD29	1:200 (F)	BD (562801)
PE Rat Anti-Mouse CD44	1:200 (F)	BD (553134)
FITC Rat Anti-Mouse CD34	1:200 (F)	BD (560238)
FITC Rat Anti-Mouse CD45	1:200 (F)	BD (553079)
Primary antibody		
Stat3 mouse mAb	1:500 (IHF) 1:1000 (WB)	CST (9139) CST (9139)
P-Stat3 mouse mAb	1:1500 (WB)	CST (4113)
OPN rabbit pAb	1:200 (IHF)	ZEN BIO (380437)
	1:1000(WB)	ZEN BIO (380437)
COL1A1 rabbit pAb	1:200 (IHF)	Abclonal (A1352)
	1:1000 (WB)	Abclonal (A1352)
GAPDH rabbit mAb	1:1000 (WB)	CST (5174)
Secondary antibody		
Anti-mouse Alexafluor-594	1:500 (IHF)	Beyotime
Anti-rabbit Alexafluor-594	1:500 (IHF)	Beyotime
Anti-mouse IgG HRP-linked Ab	1:4000 (WB)	CST (7076)
Anti-rabbit IgG HRP-linked Ab	1:4000 (WB)	CST (7074)

### Micro-CT analysis

The skeletons were scanned and analyzed using a μCT 50 system (Scanco, Brüttisellen, Switzerland) with a spatial resolution of 10 μm. For trabecular bone morphometric analysis of distal femur, the starting slice was taken where the growth plates were fully merged and extended for 100 slices proximally. The morphometric analysis was performed for bone mineral density (BMD), bone volume per tissue volume (BV/TV; %), and cortical bone thickness by μCT 50 system. The 3D reconstruction of the skeletons and cross-section images were performed by Dataviewer and CTvox (Skyscan, Belgium).

### Primary isolation and characterization of BMSCs

The femur and tibia were dissected from 8-week-old Stat3 CKO mutants and littermate controls. Bone marrow cells from long bones were flushed out by 2% fetal bovine serum (FBS, Gibco, USA) PBS. Single-cell suspensions from long bone marrow were obtained through 70-μm cell mesh and seeded on 100-mm dishes (Jet, Guangzhou, China). Non-adherent cells were removed after 3-days’ culture in alpha-MEM (Gibco, USA) supplemented with 20% FBS (Gibco, USA), 100 U·mL^−1^ penicillin, and 100 μg·mL^−1^ streptomycin (Gibco, USA), 2 mmol·L^−1^ l-glutamine (Gibco, USA), 55 μmol·L^−1^ 2-mercaptoethanol (Invitrogen, USA). Cells at passage 2 were employed for the following in vitro experiments (see Additional file 1).

The mesenchymal stem cell characterization was performed, including (a) the flow cytometry were conducted to show that these cells were positive for CD29, CD44 and were negative for CD34 and CD45 (BD Biosciences, USA), (b) osteoblastic and (c) adipogenic differentiation were applied to show the multi-lineage differentiation capacity of the primary isolated cells. The antibodies used for flow cytometry were listed in Table 2.

### Osteogenic and adipogenic differentiation of BMSCs

For osteogenic differentiation, the BMSCs were plated in 6-well plates and cultured in osteogenic induction medium containing 10 mM β-glycerol phosphate (Sigma, USA), and 50 μM ascorbic acid (Solarbio, Beijing, China) 10^−7^ M dexamethasone (Sigma, USA). After 7 days’ induction, alkaline phosphatase (ALP) expressions were detected by Alkaline Phosphatase Stain kit (Yeasen, Shanghai, China) according to the manufacturer’s instructions. Images were taken by Leica Microsystems and ImageJ was applied to quantify the stained areas. After 14-days’ osteogenic induction, the calcium deposit was evaluated by Alizarin Red S staining (Cyagen Biosciences, Guangzhou, China). Images were taken by Leica Microsystems, followed by quantification using 10% cetylpyridinium chloride (CPC) (Sigma, USA) to dissolve the stained calcium deposit. The absorbance value of the dissolved solution at 562 nm was measured by microplate spectrophotometer (Bio-Tek, UK).

Adipogenic induction medium was provided by Cyagen Biosciences, Inc. (Guangzhou, China). After 12-days’ induction according to manufacture instruction, the cultured cells were stained with oil red O solution (Cyagen, Guangzhou, China), and were observed under light microscope (Leica Microsystems).

### Western blot

Total protein of mouse hindlimbs and BMSCs was extracted using the RIPA lysis buffer (CWBIOTECH, China) containing proteinase and phosphatase inhibitors (CWBIOTECH, China), and then quantified by the BCA method (FudeBio, China). The proteins were separated by SDS-PAGE gel electrophoresis (Smart-Lifesciences, China) and transferred to a polyvinylidene fluoride (PVDF) membrane (Millipore, USA). After 1 h blocking by 5% BSA, membranes were incubated at 4 °C overnight with relevant primary antibodies. The PVDF membranes were incubated in HRP conjugated secondary antibody for 1 h at room temperature. Immunoreactivities were detected by a chemiluminescence kit (FudeBio, China). Data were normalized to GAPDH and quantified by ImageJ. The antibodies used in this study were listed in Table 2.

### Statistical analysis

The data were presented as means ± standard deviation (SD). Student's t-tests were used to compare the difference between Stat3 CKO and the control. The GraphPad Prism 7.0 software were applied for statistical analysis. The significance level was set at *P* < 0.05.

## Results

### Generation of Stat3 CKO mice

**Fig. 1:**
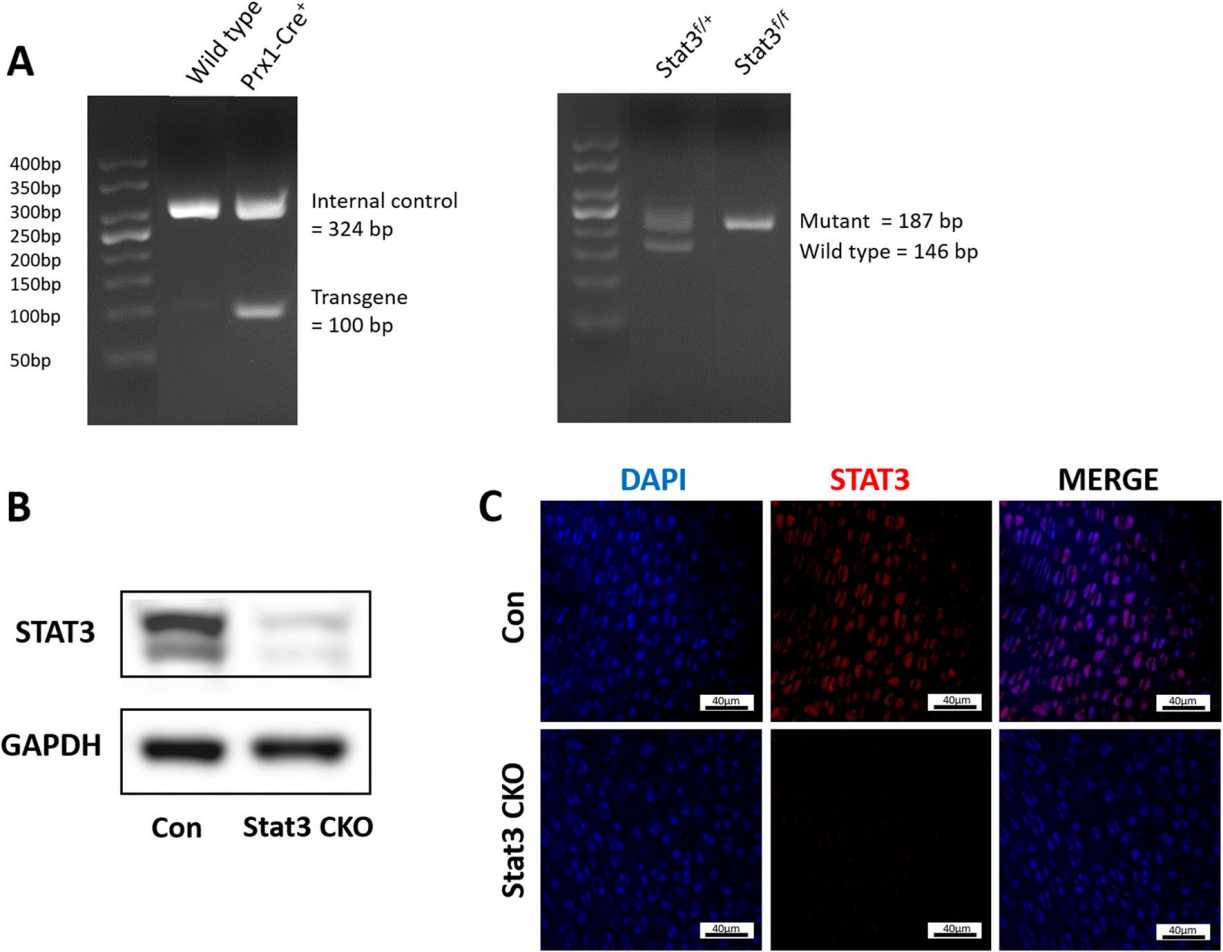
In vivo deletion of Stat3 in mesenchymal progenitors. **A** Genotyping of Stat3^f/f^; Prx1-Cre mice and Stat3^f/+^ mice tail DNA. DNA ladder: 50 bp. **B** Protein were extracted from P0 forelimbs. Western blot analysis revealed significant decrease of Stat3 protein expression in Stat3 CKO compared with control. **C** IHF staining showed the loss of Stat3 expression in bone tissues on Stat3 CKO (ulna section of P0 mice)

To investigate the function of Stat3 in skeleton development, Stat3 was deleted in mesenchymal progenitors by use of Prx1-Cre. Fig. 1A demonstrated the mouse genotypes used in this study: Prx1-Cre; Stat3^f/f^ (Stat3 CKO) and Cre negative littermate control. The deletion of Stat3 was further confirmed by western blot and IHF staining. (Fig. 1B, C). The expression of Stat3 was barely found in bone tissues of Stat3 CKO mice (Fig. 1B, C).

### Conditional deletion of Stat3 in mesenchymal progenitors led to shortened limbs and open fontanels

**Fig. 2:**
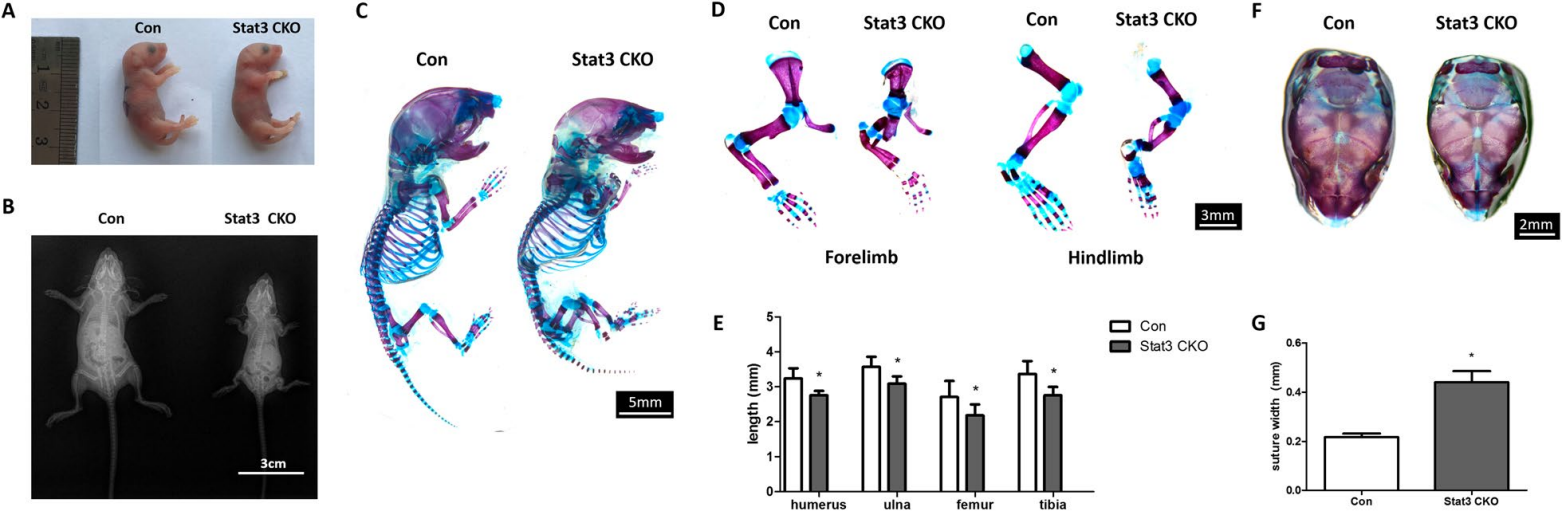
Loss of Stat3 resulted in skeletal abnormalities. **A**, **B** Ex vivo observation of control and Stat3 CKO mice at P0 and 3w. **C** Alizarin red/alcian blue staining of skeletal preparations of P0 control and Stat3 CKO mice. **D**, **E** Multiple fractures were observed in Stat3 CKO mice. Compared with the littermate control, the limbs were significantly shortened. **F**, **G** Alizarin red/alcian blue from P0 samples showed dysplasia in Stat3 CKO calvaria. Compared with the littermate control, the width of sagittal suture was significantly wider in Stat3 CKO mice. Quantitative data were obtained using the ImageJ software. n = 3, *P < 0.05 vs Con

The Stat3 CKO mice displayed shorten limbs and smaller skull in comparison with the control mice. With aging, the difference of deformed limbs and body size became more obvious between Stat3 CKO mice and their littermate control (Fig. 2A, B). Whole-mount skeletal staining by Alizarin Red and Alcian Blue demonstrated that in Stat3 CKO mice, both forelimbs and hindlimbs were distinctly shorten (Fig. 2C–E) (*P* < 0.05). The abnormalities were found in both proximal long bones (the humerus and femur) and distal long bones (the radius, ulna, tibia and fibula). Multiple fractures were observed in the forelimbs and hindlimbs of Stat3 CKO mice (Fig. 2D). As for cranium, Stat3 CKO mice showed open fontanels and more porous skull when compared with controls (Fig. 2E, G) indicating impaired intramembranous ossification.

### Stat3 CKO mice exhibited abnormal growth plate structure and impaired osteogenesis

**Fig. 3:**
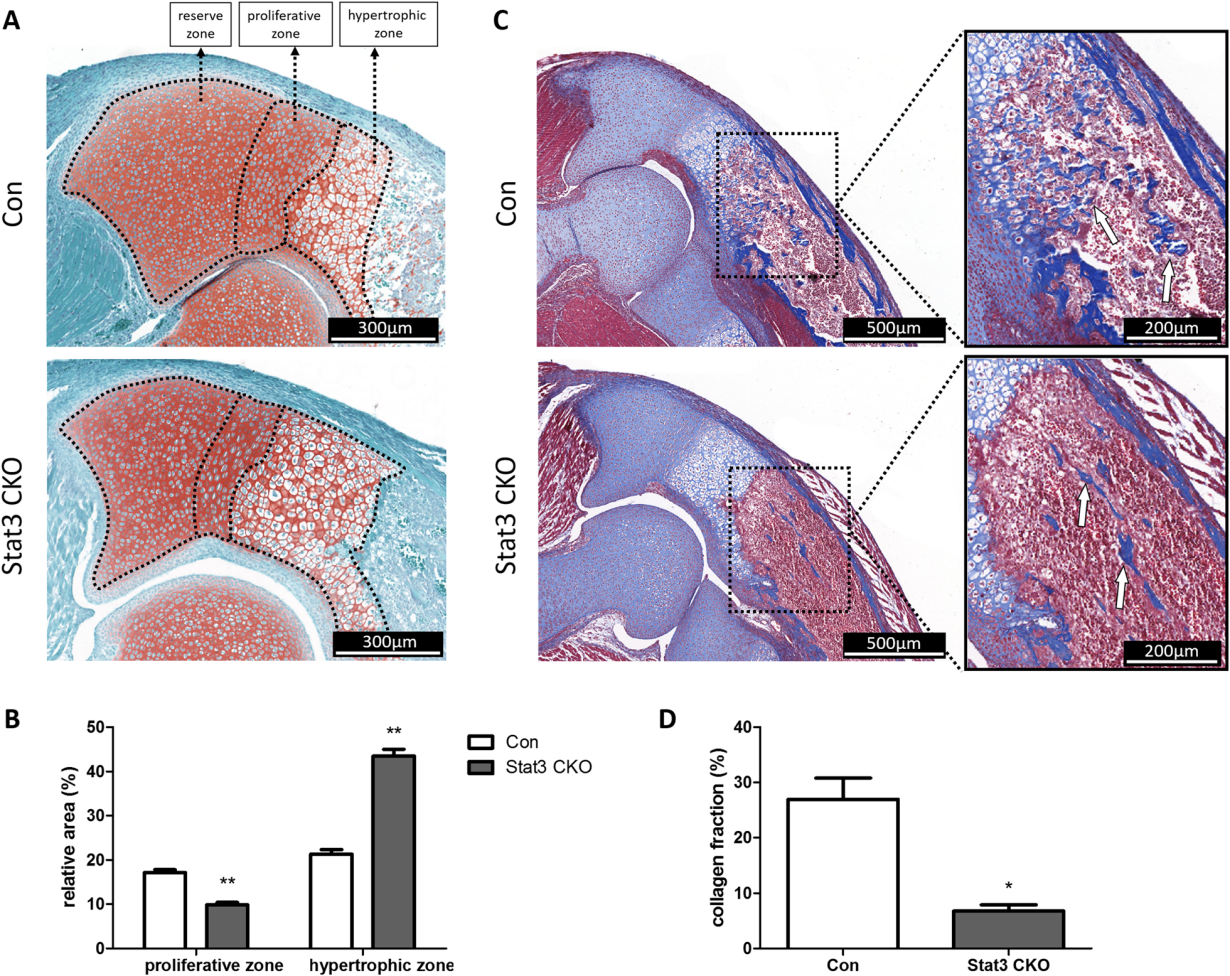
The impaired endochondral ossification was found on neonatal Stat3 CKO mice. **A**, **B** Safranin O/fast green staining of proximal ulna sections showed a decrease in the proliferative zone and an increase in the hypertrophic zone in Stat3 CKO samples when compared with the control. **C**, **D** The masson staining showed less blue-stained collagen fibers in the trabecular bone region of Stat3 CKO mice. The boxed region was enlarged in the right panel, white arrows pointing to stained collagen fibers. n = 3, *P < 0.05, **P < 0.01 vs Con

The neonatal Stat3 CKO mice showed impaired endochondral ossification. The proximal ulna sections of Stat3 CKO mice showed a decrease in the proliferative zones and an increase in the hypertrophic zones (Fig. 3A, B) (*P* < 0.05). Masson’s trichrome staining showed that plenty of collagen fiber (stained in blue by Masson) existed in the trabecular bone area and endosteal surface of cortical bone on control mice, meanwhile much less collagen was observed on the Stat3 CKO mice (Fig. 3C, D) (*P* < 0.05). IHF staining showed less COL1A1 and OPN expression at ulna proximal epiphysis and endosteal surface of cortical bone (Additional file 2: Fig. S1).

**Fig. 4:**
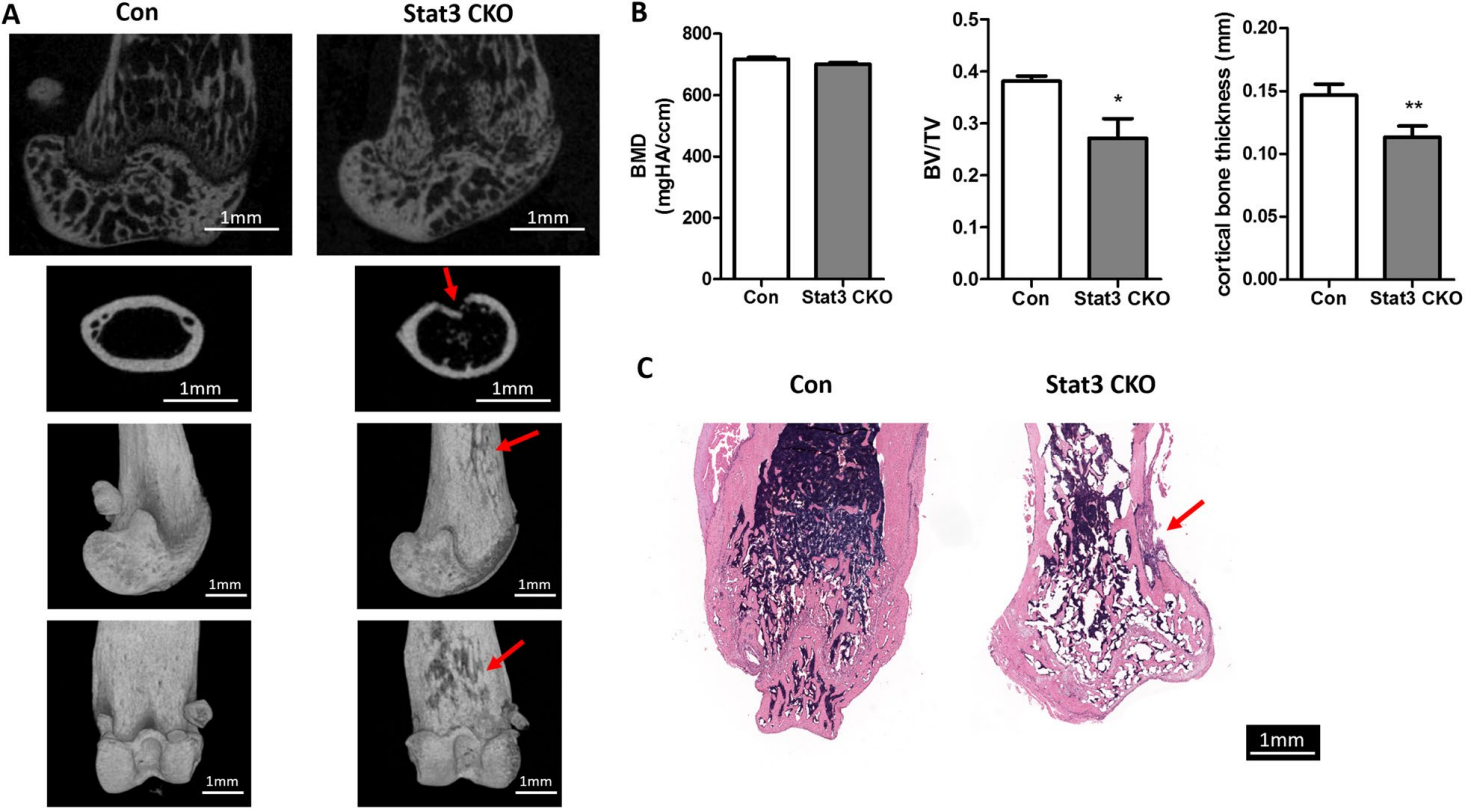
Impairment in cancellous and cortical bone in Stat3 CKO mice of 8-week-old. **A** Femurs from 8-week-old control and Stat3 CKO mice. Irregular cancellous and thinner cortical bone were observed in Stat3 CKO mice. **B** Quantification of micro-CT data showed a significantly reduced BV/TV at trabecular bone and thinner cortical bone (n = 3, *P < 0.05 vs Con). BMD: bone mineral density; BV/TV: bone volume/tissue volume. C. H&E-stained longitudinal sections of femurs from 8-week-old control and Stat3 CKO mice. Red arrows pointed to the cortical bone defect

The bone parameters were assessed for 8-week-old Stat3 CKO and control mice. The long bones of Stat3 CKO mice were shorter than littermate control (Fig. 4A). As Fig. 4A showed, Stat3 CKO mice demonstrated irregularly arranged cancellous bone in distal femur, in comparison with the well aligned cancellous bone in littermate control. The cortical bone of Stat3 CKO mice was thinner than the littermate control and some pit-like bone defect can be observed on the surface of cortical bone (Fig. 4A, B). Micro-CT analysis showed that 8-week-old Stat3 CKO mice was in significant decrease of BV/TV (*P* < 0.05), while the difference in BMD was not significant (Fig. 4B). Histology of 8-week-old Stat3 CKO femur was in accordance with micro-CT results. (Fig. 4C).

### Primary isolation and characterization of BMSCs

**Fig. 5:**
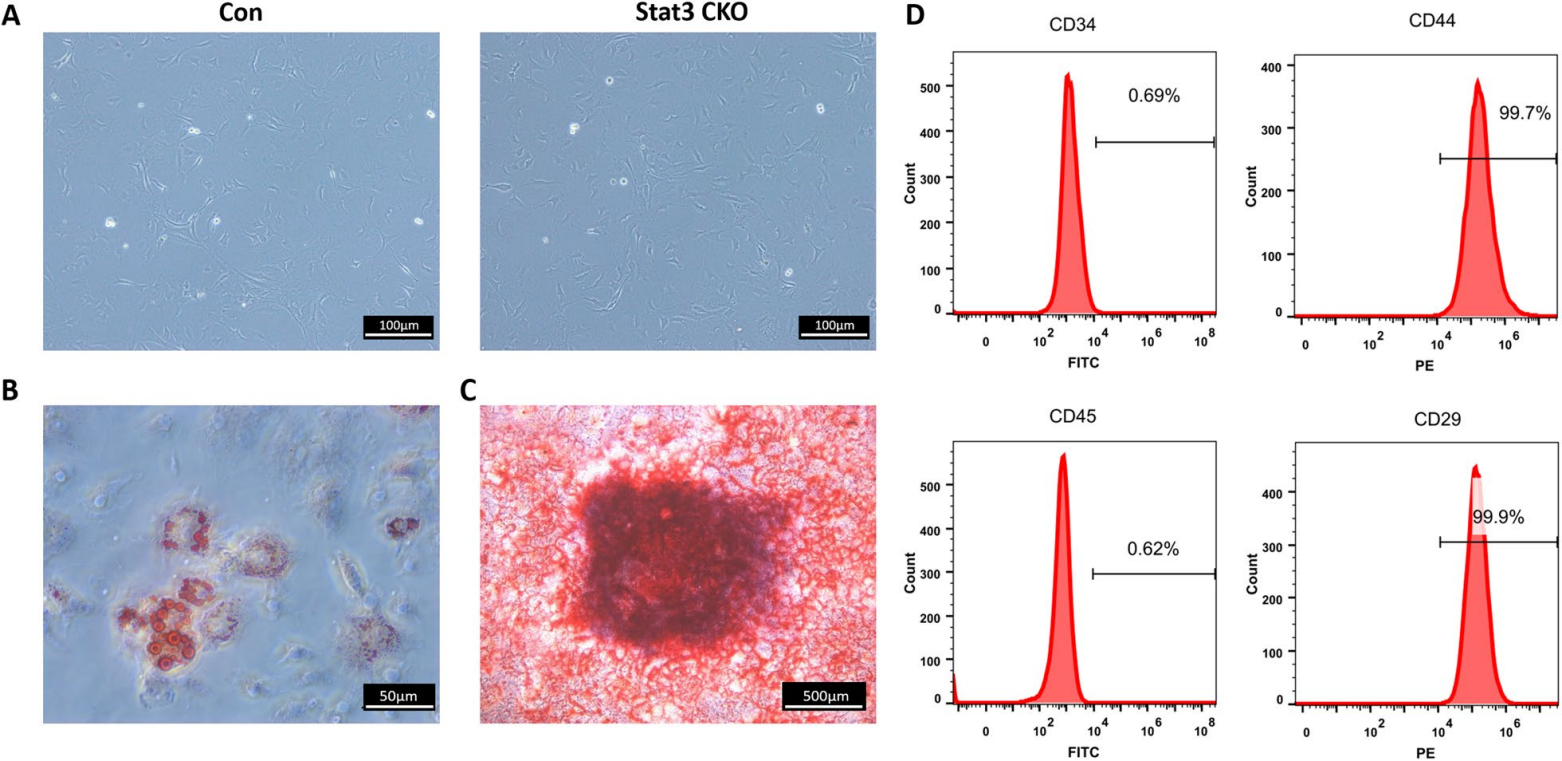
Isolation and characterization of bone marrow mesenchymal stem cells (BMSCs). **A** Primary isolation of bone marrow mesenchymal stem cells (BMSCs) (5-week-old mouse, passage 2). **B** Oil red O staining were performed after adipogenic induction for 12d. **C** Alizarin red staining were performed after osteogenic induction for 14d. D. Flow cytometry analysis of cell surface markers of CD29, CD44, CD34, and CD45 in primary isolated BMSCs

Given that the skeletal abnormalities in Stat3 CKO mice might be owing to defects in bone formation, in vitro experiments were carried out to investigate the cellular mechanism associated with the phenotype. BMSCs were isolated from Stat3 CKO mice and littermate control respectively (Fig. 5A). The primary isolated BMSCs were able to differentiate into adipogenic (Fig. 5B) and osteogenic (Fig. 5C) linage cells, which supported their capacity of multi-lineage differentiation (Fig. 5B, C). In the flow-cytometry, the primary isolated BMSCs were positive for stem cell markers of CD29 and CD44 while negative for hematopoietic markers of CD34 and CD45 (Fig. 5D).

### Loss of Stat3 resulted in decreased osteogenesis of BMSCs

**Fig. 6:**
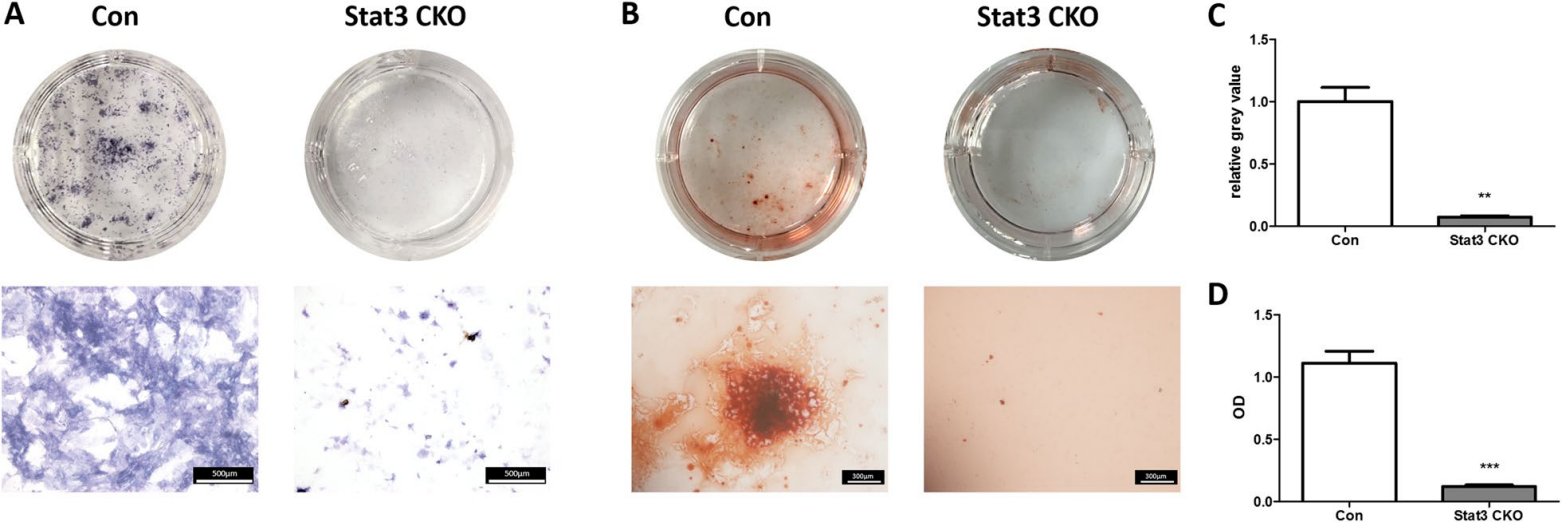
The decreased ALP expression and mineralization were found on Stat3 CKO BMSCs. **A** The alkaline phosphatase (ALP) staining was performed at 7d of osteogenic induction. **B** The alizarin red staining (ARS) was done at 14d of osteogenic induction. **C** Quantitative data of ALP staining were obtained by ImageJ software. **D** Alizarin Red concentrations were determined by a quantitative destaining procedure using cetylpyridinium chloride (CPC). n = 3, **P < 0.01, ***P < 0.001 vs Con

**Fig. 7:**
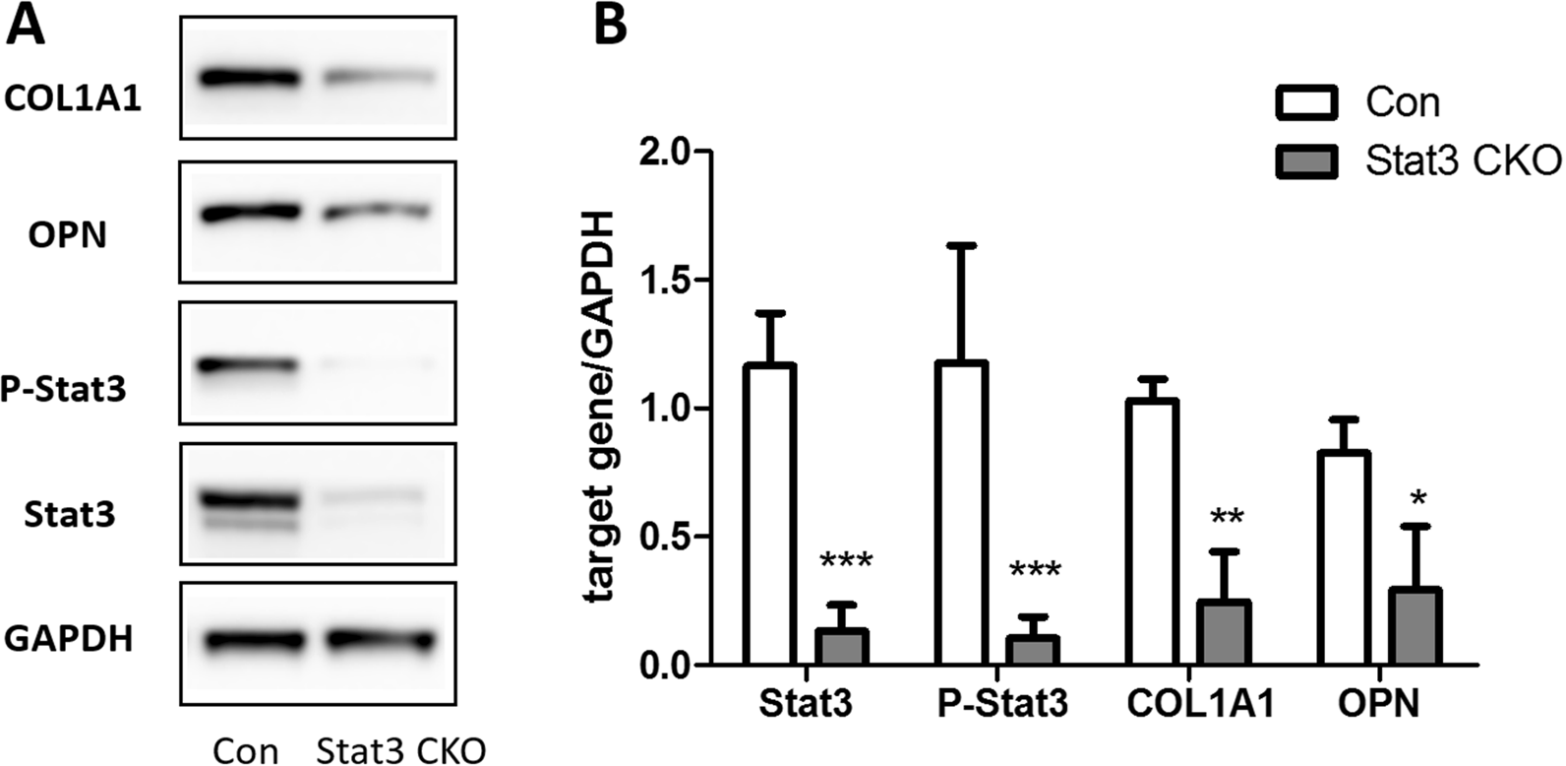
After osteogenic induction for 14 days, Stat3 CKO BMSCs expressed less osteogenic makers when compared to the control. **A** BMSCs expressions of osteogenic markers were assessed by western blotting. **B** Quantitative analysis of Stat3, P-Stat3 and osteogenic markers (COL1A1 and OPN) expression levels. n = 3. *P < 0.05, **P < 0.01, ***P < 0.001 vs Con

After osteogenic induction for 7d, Stat3 CKO BMSCs showed a pronounced decrease of alkaline phosphatase (ALP) staining intensity, compared with the control (*P* < 0.05) (Fig. 6A). According to the alizarin red (ARS) staining, much less calcium deposits were found in Stat3 CKO BMSCs after 14d osteogenic induction, *versus* in control BMSCs (*P* < 0.05) (Fig. 6B). Quantitative measurements of ALP intensity and Alizarin Red concentration were displayed in Fig. 6C, D. In response to osteogenic induction, Stat3 CKO BMSCs showed a decreased expression of OPN and COL1A1 when compared with the control (Fig. 7A). Further quantitative analyses of protein expression revealed that the decrease of OPN and COL1A1 in Stat3-deficient BMSCs was statistically significant (*P* < 0.05) (Fig. 7B).

### Stat3 could activate the transcription of mouse *opn* and *col1a1* genes

ChIP-seq peaks were found around the transcription start sites (TSS) of both *opn* and *col1a1* genes, indicating that there were putative binding sites of Stat3 on these genes (Additional file 3: Fig. S2). For *opn* gene, the ChIP-seq peak of Stat3 fell right on TSS, and for *col1a1* gene, the peak was 780 bp away from the TSS (Additional file 3: Fig. S2).

## Discussion

In the present study, the severe bone abnormality was observed in Stat3 CKO mice, including shortened limbs, multiple fractures, and reduced bone volume. In vitro study showed that the osteogenic differentiation was reduced in Stat3-deficient BMSCs when compared with the control.

Skeletal development is an orchestrated process comprising intramembranous and endochondral ossification, which both begin with condensation of mesenchymal progenitors [16]. During intramembranous ossification, mesenchymal progenitors directly differentiate into osteoblasts to form mineralized bone. However, during endochondral ossification, mesenchymal progenitors differentiate into chondrocytes and perichondria cells [17]. Then, the chondrocytes undergo proliferation and hypertrophy. The hypertrophic chondrocytes express Indian hedgehog (Ihh) to induce the initial osteoblast differentiation [18]. Growth plate chondrocytes can be replaced by bone and careful coordination of this process are essential for endochondral ossification [17]. In the present study, abnormal growth plate structure was found in Stat3 CKO mice. In the proximal ulna growth plate of Stat3 CKO mice, the proliferative zone was narrower and the hypertrophic zone was wider than the control. The proliferation of chondrocytes contributes greatly to the linear elongation of long bones [19, 20]. In our study, the shorten limbs in Stat3 CKO mice may result from the decreased proliferative potential of chondrocytes in growth plates. According to Hall et al., the deletion of Stat3 in mesoderm also resulted in an enlargement of hypertrophic chondrocyte zone and a decrease of limb length. The expression of Sox9, a critical factor in the regulation of chondrogenesis and subsequently endochondral ossification, was directly regulated by Stat3 [9]. It is known that the hypertrophic chondrocytes express enzymes such as matrix metalloproteinase 13 (MMP13) and undergo apoptosis accompanied with vascular invasion. The apoptosis chondrocytes are replaced by mesenchymal stromal cells that differentiate into osteoblasts to produce bone matrix proteins, the most of which was type 1 collagen [17, 20, 21]. In our study, Stat3 CKO mice showed less collagen fiber in the trabecular bone area when compared with control, especially around the chondro-osseous front. This phenomenon might imply that loss of Stat3 can impair the differentiation of mesenchymal stromal cells into osteoblasts, and the ability of osteoblasts to produce bone collagen which is key to form bone matrix.

Stats family has various functions in mammalian development [22, 23]. Among Stats family, Stat3 was believed to be profoundly associated with skeleton homeostasis [24]. In vivo studies have shown that malfunction of Stat3 led to distinguishable bone abnormality in skeletal system [5, 9, 11]. Our previous study demonstrated that overexpression of Stat3 rescued the decreased osteoblast differentiation of Ror2-knockdown mBMSCs, suggesting Stat3 could be the downstream molecule of Ror2 [25]. Ror2 was widely accepted as a transmembrane Wnt receptor in canonical and non-canonical Wnt signaling pathway [26–28]. Mice with conditional knockout of Ror2 in mesenchymal progenitors exhibited shorten limbs [25], which was similar to the phenotype of Stat3 CKO mice in the present study. Moreover, the previous study showed a decrease in the width of the proliferative zones and an increase in the width of the hypertrophic zones in the humerus of Ror2^−/−^ mice, which was highly consistent with our findings in the proximal ulna of Stat3 CKO mice [29]. This similarity in phenotype of the above studies not only reinforced the importance of Wnt signaling pathway in skeleton development, but also indicated the involvement of Stat3 in Ror2-mediated Wnt signaling. Furthermore, a recent study reported the effect of Stat3 on osteoblastic differentiation through Wnt/β-catenin signaling by use of the conditional knockout of Stat3 in mesenchymal progenitors (Prrx1Cre; Stat3^c/c^ mice) [30]. As expected, the in vivo bone defects by loss of Stat3 were similar to our study. The skeletal phenotype of Stat3 CKO was partially rescued by enhancing Wnt/β-catenin signaling [30]. Taken together, Stat3 could be an important regulator in both canonical and non-canonical Wnt signaling for bone formation and maintenance.

The in vitro expression of osteogenic markers (OPN and COL1A1) was found diminished in response to Stat3-dificiency. OPN is one of the major non-collagenous proteins that plays key roles in determining bone morphology [31]. Bioinformatics analysis found Stat3 transcription factor binding site (TFBS) in both human and mice OPN gene, indicating that Stat3 might play a role in the transcriptional regulation of OPN gene [6]. The other down-regulated protein in Stat3 CKO BMSCs was COL1A1, which contributed importantly to bone matrix [32]. Fan and colleagues found that in the human hepatic stellate LX-2 cells, COL1A1 might act as a downstream gene in Jak1/Stat3 signaling pathway [33]. Our ChIP-seq analysis showed Stat3 could bind to the transcription start site of *opn* and *col1a1*, but the exact interaction between OPN, COL1A1, and Stat3 needs to be further explored. Nevertheless, in our study, Stat3 deficiency resulted in decreased expression of both collagenous (COL1A1) and non-collagenous (OPN) protein in vitro, which in turn, impaired the osteogenic differentiation of BMSCs and less mineralization of the cells was found.

Besides OPN and COL1A1, there were some other genes that could interact with Stat3 according to literatures. Studies have discovered that activated Stat3 could bind to the promoter of several osteogenesis related genes, such as Runx2 [34] and OCN [35]. Xu et al. reported that icariin could regulate OCN transcription through Stat3 in mBMSCs [35]. More specifically, P-Stat3 interacted with Runx2, forming a complex to bind to the OCN promoter and enhanced mRNA expression of OCN [35]. However, Dalagiorgou et al.considered that in mechanically stimulated osteoblasts, P-Stat3 directly bonded to the Runx2 promoter and upregulated mRNA level of Runx2 [34]. In addition to osteogenic markers, Stat3 could also play an important role in chondrogenesis by direct regulating the Sox9 expression [9]. The specific transcriptional role of Stat3 might be different under particular circumstances and further research was needed to elucidate the mechanism of Stat3 in BMSC-mediated osteogenesis.

In conclusion, Stat3 was essential for osteogenesis of mesenchymal progenitors in vivo and in vitro. Stat3 CKO mice showed severe skeletal abnormality and Stat3-deficient BMSCs was presented with reduced osteogenic differentiation ability, likely in part by regulating the expression of OPN and COL1A1.

## Supplementary Information

**Additional file 1:** .

**Additional file 2:Fig. S1.** Decreased expression of COL1A1 and OPN in P0 Stat3 CKO mice (P0) compared with the control.

**Additional file 3:Fig. S2.** Stat3 could activate the transcription of mouse col1a1 and opn genes.

## Data Availability

All datasets used and/or analyzed during the current study are available from the corresponding author on reasonable request.

## Abbreviations

Stat3: Signal transducer and activator of transcription 3
CKO: Conditional knockout
PCR: Polymerase chain reaction
IHC: Immunohistochemistry
BMSCs: Bone marrow mesenchymal stem cells
FBS: Fetal bovine serum
BSA: Bovine serum albumin
Ror2: Receptor tyrosine kinase like orphan receptor 2
OSX: Osterix
OPN: Osteopontin
COL1A1: Collagen I
OCN: Osteocalcin
Runx2: Runt-related transcription factor-2
