# Supplementary material for: Loss of signal transducer and activator of transcription 3 impaired the osteogenesis of mesenchymal progenitor cells in vivo and in vitro
Source: Cell Biosci. 2021 Sep 8;11:172. doi: 10.1186/s13578-021-00685-3 (PMC8424822; doi:10.1186/s13578-021-00685-3)
Supplement: Supplementary file 1 — Additional file 1: . [file 13578_2021_685_MOESM1_ESM.docx]

**Loss of signal transducer and activator of transcription 3 impaired the osteogenesis of mesenchymal progenitor cells *in vivo* and *in vitro***

**Zijing Huang^1,2#^, Jingyi Feng^1,2#^, Xin Feng^1,2^, Laiting Chan****^1,2^, Jiarui Lu^1,2^, Lizhen Lei^1,2^, Zhuwei Huang^1,2^, Xiaolei Zhang^1,2^***

**Supplementary materials and methods**

Chromatin immunoprecipitation-sequencing (ChIP-seq) analysis

Fastq files from ChIP-seq were processed by the pipeline of AQUAS Transcription Factor and Histone (https://github.com/kundajelab/chipseq_pipeline). Briefly, sequencing tags were mapped against the *Mus musculus* reference genome by using BWA 0.7.1551. ChIP-seq peaks were identified using MACS2.0 (Feng J *et al*, 2012) by comparing ChIP library to input DNA library. All sequencing tracks were visualized by Intergrative Genomics Viewer (IGV) genome browser (Thorvaldsdóttir H *et al*, 2013).

**Supplementary figures**

**Fig. S1 Decreased expression of COL1A1 and OPN in P0 Stat3 CKO mice (P0) compared with the control.** IHF staining showed less COL1A1 and OPN expressed around ulna proximal epiphysis and endosteal surface cortex.

**Fig. S2** **Stat3 could activate the transcription of mouse *col1a1* and *opn* genes.**

Red boxes and bars indicate the ChIP-seq peaks of Stat3 around the transcription start sites (TSS). The distance from the peak to transcription start site (TSS) is shown below.

**References**

Feng J, Liu T, Qin B, Zhang Y, Liu XS. Identifying ChIP-seq enrichment using MACS. Nat Protoc. 2012;7(9):1728-40.

Thorvaldsdóttir H, Robinson JT, Mesirov JP. Integrative Genomics Viewer (IGV): high-performance genomics data visualization and exploration. Briefings in bioinformatics. 2013;14(2):178-92.
