# Supplementary figures and images for: Loss of signal transducer and activator of transcription 3 impaired the osteogenesis of mesenchymal progenitor cells in vivo and in vitro
Source: Cell Biosci. 2021 Sep 8;11:172. doi: 10.1186/s13578-021-00685-3 (PMC8424822; doi:10.1186/s13578-021-00685-3)

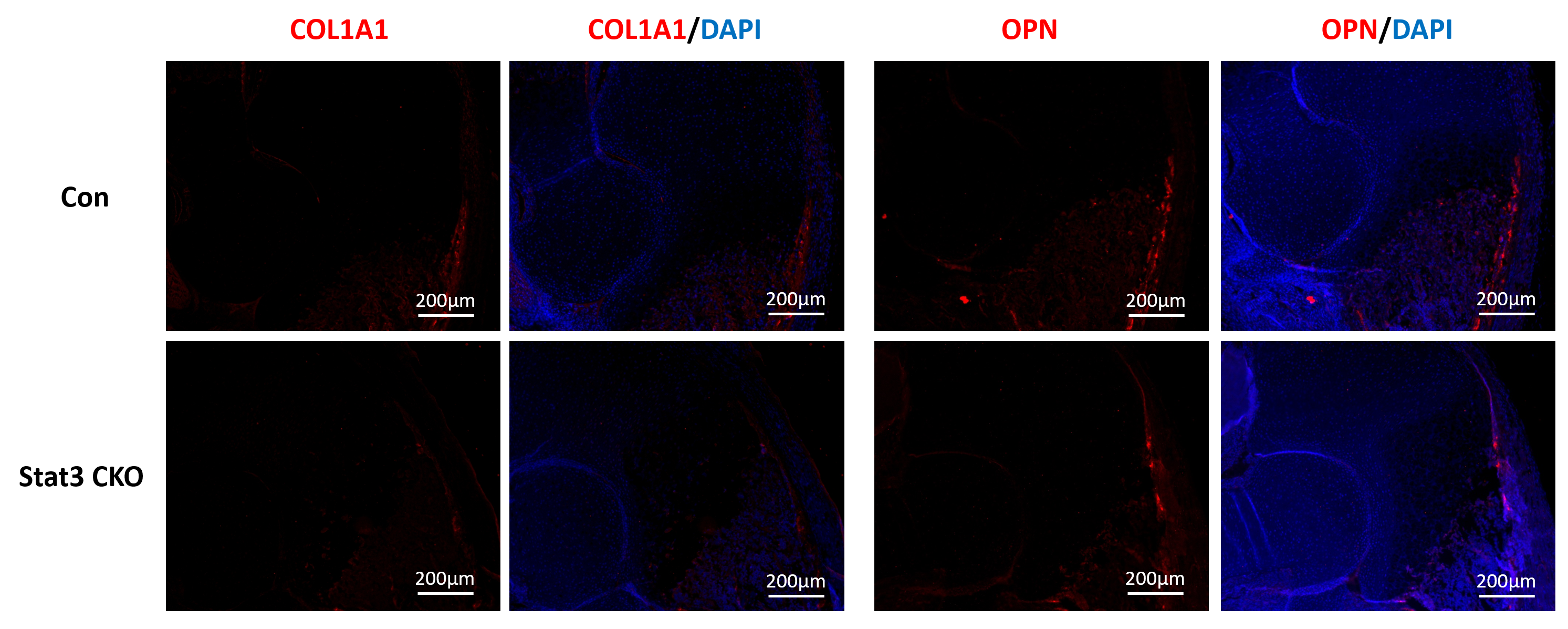

Supplement: Supplementary file 2 — Additional file 2:Fig. S1. Decreased expression of COL1A1 and OPN in P0 Stat3 CKO mice (P0) compared with the control. [file 13578_2021_685_MOESM2_ESM.tif]

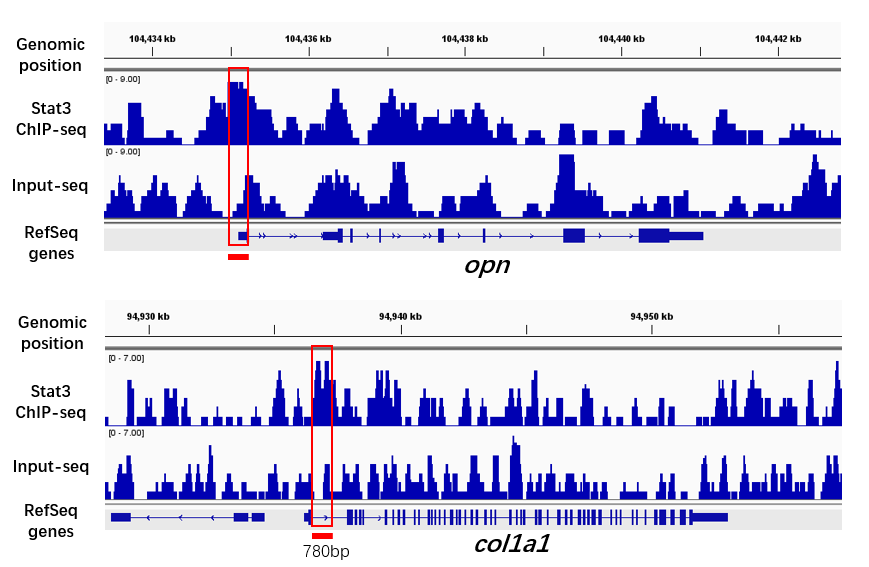

Supplement: Supplementary file 3 — Additional file 3:Fig. S2. Stat3 could activate the transcription of mouse col1a1 and opn genes. [file 13578_2021_685_MOESM3_ESM.tif]
